# Development of the indirect flight muscles of *Aedes aegypti*, a main arbovirus vector

**DOI:** 10.1186/s12861-021-00242-8

**Published:** 2021-08-26

**Authors:** Antonio Celestino-Montes, Salvador Hernández-Martínez, Mario Henry Rodríguez, Febe Elena Cázares-Raga, Carlos Vázquez-Calzada, Anel Lagunes-Guillén, Bibiana Chávez-Munguía, José Ángel Rubio-Miranda, Felipe de Jesús Hernández-Cázares, Leticia Cortés-Martínez, Fidel de la Cruz Hernández-Hernández

**Affiliations:** 1 Departamento de Infectómica y Patogénesis Molecular, Centro de Investigación y de Estudios Avanzados del IPN, Av. Instituto Politécnico Nacional # 2508, San Pedro Zacatenco, G. A. Madero, 07360 México City, México; 2 Centro de Investigación Sobre Enfermedades Infecciosas, Instituto Nacional de Salud Pública, Av. Universidad # 655, Santa María Ahuacatitlán, 62100 Cuernavaca, Morelos México; 3 Departamento de Biología Celular, Centro de Investigación y de Estudios Avanzados del IPN, Av. Instituto Politécnico Nacional # 2508, San Pedro Zacatenco, G. A. Madero, 07360 México City, México

**Keywords:** *Aedes aegypti*, Indirect flight muscles, Dorsal—longitudinal muscles, Dorsal–ventral muscles, Muscle development, Myoblast, Myotube, Myofibril, Sarcomere

## Abstract

**Background:**

Flying is an essential function for mosquitoes, required for mating and, in the case of females, to get a blood meal and consequently function as a vector. Flight depends on the action of the indirect flight muscles (IFMs), which power the wings beat. No description of the development of IFMs in mosquitoes, including *Aedes aegypti*, is available.

**Methods:**

*A. aegypti* thoraces of larvae 3 and larvae 4 (L3 and L4) instars were analyzed using histochemistry and bright field microscopy. IFM primordia from L3 and L4 and IFMs from pupal and adult stages were dissected and processed to detect F-actin labelling with phalloidin-rhodamine or TRITC, or to immunodetection of myosin and tubulin using specific antibodies, these samples were analyzed by confocal microscopy. Other samples were studied using transmission electron microscopy.

**Results:**

At L3–L4, IFM primordia for dorsal-longitudinal muscles (DLM) and dorsal–ventral muscles (DVM) were identified in the expected locations in the thoracic region: three primordia per hemithorax corresponding to DLM with anterior to posterior orientation were present. Other three primordia per hemithorax, corresponding to DVM, had lateral position and dorsal to ventral orientation. During L3 to L4 myoblast fusion led to syncytial myotubes formation, followed by myotendon junctions (MTJ) creation, myofibrils assembly and sarcomere maturation. The formation of Z-discs and M-line during sarcomere maturation was observed in pupal stage and, the structure reached in teneral insects a classical myosin thick, and actin thin filaments arranged in a hexagonal lattice structure.

**Conclusions:**

A general description of *A. aegypti* IFM development is presented, from the myoblast fusion at L3 to form myotubes, to sarcomere maturation at adult stage. Several differences during IFM development were observed between *A. aegypti* (Nematoceran) and *Drosophila melanogaster* (Brachyceran) and, similitudes with *Chironomus* sp*.* were observed as this insect is a Nematoceran, which is taxonomically closer to *A. aegypti* and share the same number of larval stages.

**Supplementary Information:**

The online version contains supplementary material available at 10.1186/s12861-021-00242-8.

## Background

*Aedes aegypti* (Diptera: Nematocera) is a major vector of arboviruses as dengue, chikungunya, Zika, and yellow fever, affecting millions of people annually in tropical and sub-tropical regions, representing important public health problems [1–5]. Female blood feeding, a requirement for reproduction, is determinant for pathogens transmission from infected to uninfected hosts and depends on flight capacity. At present, no effective vaccines and drugs against these pathogenic agents exist, and the most effective strategies for disease control are those directed against mosquito vectors [6]. Furthermore, *A. aegypti* adaptation to urban areas, environmental changes, and the rising of insecticide-resistant vector populations, make control increasingly difficult [7–9]. Thus, the importance of studying the biology of these mosquitoes, might provide insights for new strategies to interrupt disease transmission [6].

In the adult stage of Dipterans order to which the mosquito and the fruit fly belong, the indirect flight muscles (IFMs) are outstanding because they are the responsible for wing beating [10–12]. Located in the thorax, IFMs are not directly attached to the wings but to the thoracic exoskeleton and, interestingly, their contraction frequency, that supplies the power for flight and the beating frequency, is higher than those of the nerve impulses, condition called asynchrony [13, 14]. IFMs, activated mechanically by stretch, generates contraction of the thorax through connections to the cuticular exoskeleton, creating wing movements and, high oscillatory power required to flight [15, 16], which is essential in harmonic convergence during courtship [17]. This is a dynamic process where male and female form aerial swarms, adjusting their wing beat frequency and flight tone for successful mating and survival [18, 19].

In the model organism *Drosophila melanogaster*, IFMs are constructed during metamorphosis, following sequential morphological and biochemical changes [20, 21]. *D. melanogaster* IFMs have six dorsal—longitudinal muscles (DLM) and seven dorsal–ventral muscles (DVM) per hemithorax, with opposite orientation and attachment sites, which fill the adult fly thorax [20]. Myogenesis is a complex process that includes myogenic cell determination, diversification of muscle precursors, the proliferation of myoblasts, migration, physical alignment, adhesion and fusion and, finally, differentiation of functional muscles [21–26]. Two periods of myogenesis have been described during *D. melanogaster* development, the first ensue during embryogenesis to form the muscles required for larval movement; the second initiates in late third larval stage and continues during pupation to form the adult muscles including IFMs [21, 22, 27]. IFMs are generated by adult muscle precursor cells (AMP) associated to wing disc, which migrate to specific locations [28–34]; DVM formation is initiated by de novo fusing of founder cells (FCs) and fusion-competent myoblasts (FCM) to form syncytial myotubes. DLM are formed through multiple rounds of fusion of FCM to three persistent larval muscles templates that escape histolysis, the longitudinal oblique muscles (LOM), that function as FCs [35, 36] and prime syncytial myotubes formation [37, 38]. Then, myotubes attach to the tendon cells acquiring mechanical tension, evolve into myofibers, which enter to a hypertrophic phase of growth, with massive expression of structural genes and assembly of myofilaments that evolve to myofibrils and finally, the differentiation of sarcomeres (myofibrillogenesis and sarcomerogenesis) [39–42].

Although early studies have partially shown the morphology of the flight muscles of *A. aegypti* in the adult stage [10, 43, 44], myogenesis process has not been studied in this mosquito. Here, using histochemistry, immunolocalization and confocal and electron microscopy, we show a general IFM development view of *A. aegypti* mosquito throughout the third and fourth larval instars, and pupal and adult stages. We show the formation of DLM and DVM primordia, images suggest the myoblast fusion during myogenesis which leads to myofibrils growth and the formation of mature sarcomeres. Several differences during time course of IFM development were observed between *A. aegypti* and *D. melanogaster* and, similarities with *Chironomus* sp*.*

## Results

### IFMs precursors in 3 and 4th instar larvae

**Fig. 1:**
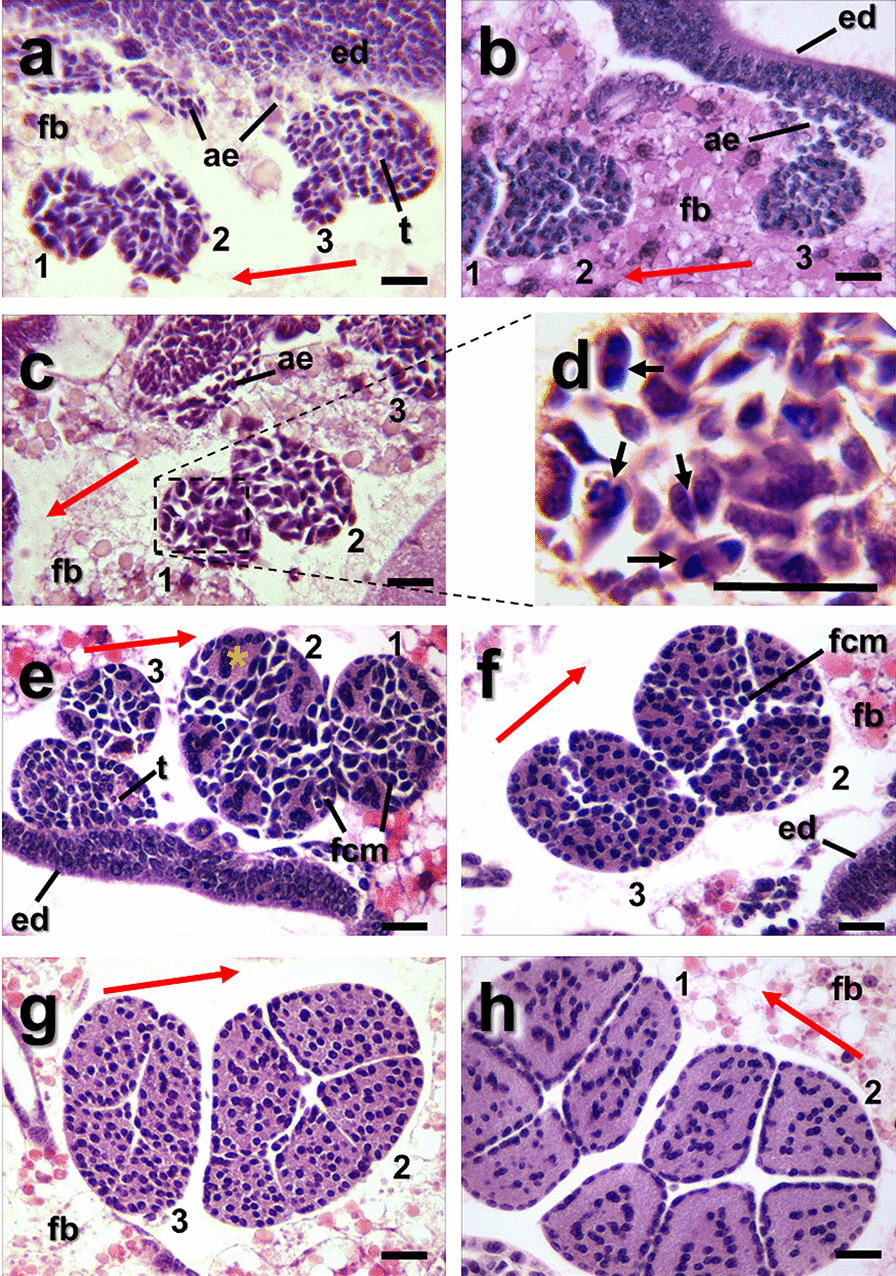
IFM precursors in *A. aegypti* larvae, DVM. **a**–**c** Coronal sections of late L3 thorax hematoxylin and eosin stained showing three myoblast nests transversely cut. **a**, **b** Those three myoblast clumps per hemithorax were labeled as 1, 2 and 3, which correspond to future formation of DVM I, DVM II and DVM III, respectively. Adepithelial cells (ae) next to the basal side of the imaginal wing disc epithelium (ed), are observed. **c**, **d** Myoblast morphology during L3 instar. Inset (**d**) showing diversity of morphology among such as “teardrop”, spindle shapes and dividing cells (arrows). **e**, **f** In early fourth-instar larvae developing DVM, initiate de novo formation by fusion of FCM to FC establishing multinucleated areas (yellow asterisk in e) to form primordial myotubes surrounded by FCM. Fascicles 1, 2 and 3 show four, five and four (or five) fusion sites, respectively, showing large nuclei in the center of each primordial myotubes. **f** The myotubes continue their development to form multinucleate myotubes, where fascicles 2 and 3 have 5 and 4 myotubes, respectively, with abundant myoblasts among them. **g**, **h** During late fourth-instar larvae the myotube formation is almost complete, where most frequently, fascicles 1, 2, 3 are composed by 4, 5, 4 myotubes, respectively. Syncytial myotubes can be observed with homogeneous nuclei distribution (**g**) or aligned in rows (**h**). At this stage, scarce FCM were observed. Red arrows point toward cephalic side of the larva. *ae* adepithelial cells, *ed* wing epithelial disc, *fcm* fusion competent myoblast, *fc* founder cells, *fb* fat body, *t* tergal depressor trochanter. Scale bar: 50 µm

In the dipteran’s thorax there are two groups of IFMs, which are the most prominent thoracic muscles, corresponding to the DLM and DVM. To study the IFM development of *A. aegypti* mosquito, we performed hematoxylin–eosin (HE) histochemical (HC) analysis of thoraces of third (L3) and fourth (L4) larval instars. In coronal sections from late L3, we observed, in the expected position for DVM, based on *D. melanogaster* location [20], three well-defined structures (likely muscle primordia) per hemithorax, transversally cut and numbered from anterior to caudal location as 1, 2 and 3 (Fig. 1a–c), formed by groups of cells with a similar morphology to *D. melanogaster* precursor myoblasts, each enveloped by a membrane (Fig. 1a–c). In this region, myoblast clusters are close to cells corresponding by location and morphology to possible adepithelial cells (ae) and presumably the epithelium of wing imaginal disc (ed) and fat body (fb) cells were also observed. The location and morphological characteristics of the three primordia corresponded to DVM I, DVM II and DVM III were labeled as DVMs 1, 2 and 3 (Fig. 1a–c and Additional file 1: Fig. S1a, b). The imaginal myoblasts seem to originate a population of cells which showed different shapes very similar to those described in *D. melanogaster* as “teardrop-shaped” and “spindle-shaped” unfused myoblasts, and others which have their nuclei in division, suggesting that they could be fusion competent myoblasts (FCM) (Fig. 1d, arrows), which could be migrating to the region where muscle primordia of DVM will develop.

In L4 the primordial DVMs have a specific number of myotubes in formation (Fig. 1e–h), majorly, the DVM 1, DVM 2 and DVM 3 have 4, 5 and 4 primordial myotubes, respectively. In addition, other structures were recognizable as the wing disc epithelium (ed) and a possible primordium of tergal depressor of the trochanter muscle (TDT or jump muscle), intensely stained, where the putative myoblasts are in a single compartment, as correspond to the precursor of a tubular muscle (Fig. 1a, e).

Subsequently, in sections of thorax from early L4 instar, the presumptive structures corresponding to DVM primordia were seen in the same positions as in L3 and they were larger than the previous ones (Fig. 1e, f). Inside an early L4 primordium, what seem to be the founder cells (FC) organize in discrete syncytial tubes (myotubes) with large intensely stained nuclei at the center with a clear cytoplasm and, among the myotubes there are abundant individual cells corresponding to FCM (Fig. 1e). In a next step, during L4, the number of nuclei inside the myotubes increased and the number of free myoblasts diminished (Fig. 1f). In late L4 instar, the DVM primordia maintained the same number of myotubes as in instar L3 and the interstitial FCMs diminished and finally disappeared (Fig. 1g, h). The nuclei inside myotubes organized and formed rows (Figs. 1h, 3 and Additional file 2: Fig. S3) suggesting the evolution of DVM and their transition to myofibers.

Despite DVM 1, 2 and 3 have 4, 5 and 4 myotubes, respectively, as the most frequent numbers (Fig. 1e–h), we found that eventually these numbers can fluctuate. In one selected  sample, at both sides of the thorax the DVMs showed 4, 5 and 4 fascicles, as expected (Additional file 1: Fig. S1a), while other thorax sample showed, at the right side, the DVM 1 and DVM 2 showed 5 and 6 fascicles, respectively (Additional file 1: Fig. S1b).

### The DLM formation

**Fig. 2:**
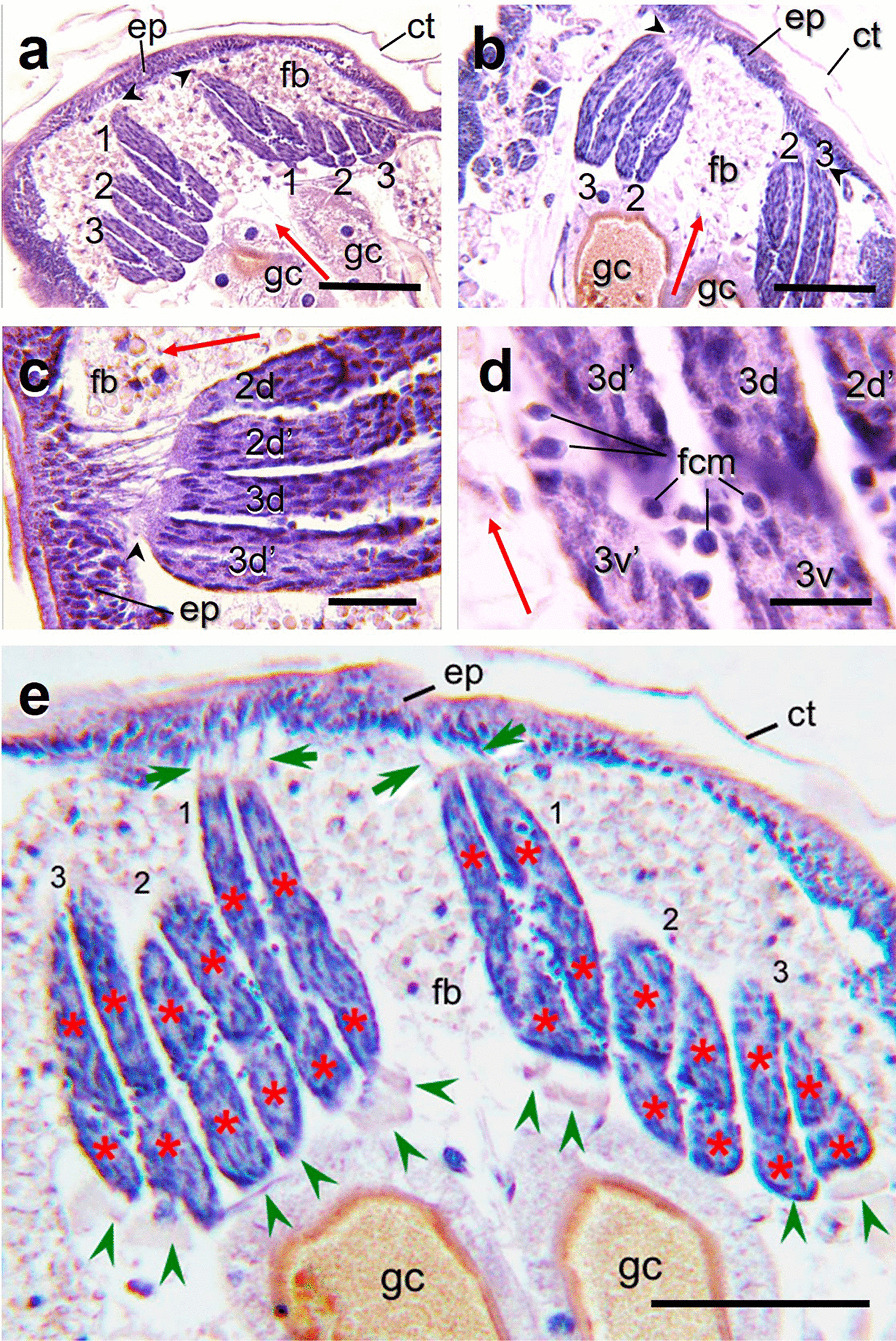
Dorso-coronal sections of the thoracic region of late L4 show fascicles corresponding to DLMs. Myotubes in each fascicle are showed in oblique sections. **a**, **b** Each DLM fascicle (three each side, right and left, labeled as 1, 2 and 3) is formed by four myotubes. **a** At this section level fascicles 1 are attached by the anterior side to the thoracic epithelium in upper thorax (black arrowheads). **b** In this plane attachment of DLM fascicles numbered 2 and 3 to the thoracic epithelium is to the lower thorax (black arrowheads) and fascicles 1 are out of focus in the histological section. **c** Detail of attachment region of DLM myotubes 3 and 2, where myotendinous junctions (MTJ) are observed (black arrowhead; d and d′, dorsal). **d** Detail of fascicle 3 where possible fusion competent myoblasts (fcm) are observed between four myotubes. **e** DLM primordia in the thoracic region of L4 instar. At this oblique section the three fibers per hemithorax are captured (numbered 1, 2, 3), each fascicle formed by four fibers (red asterisks). Those fascicles corresponding to the LOMs or scaffold myotubes, possibly in splitting process. Several myoblasts among fibers are observed (spots among fibers).  Myotendinous junctions (MTJ) formed between front side end of myotubes, and thoracic epithelial cells are shown (green arrows). The posterior end of myotube is suspended in the hemocoel and structures suggesting the start of MTJ formation are seen (green arrowheads). Red arrows point to the cephalic side. *ct* cuticle, *ep* epidermis, *fcm* fusion competent myoblasts, *fb* fat body, *gc* gastric caeca. Scale bars: **a**, **b**, **e** 500 µm; **c** 150 µm; **d** 100 µm

In respect to DLM, it is known that, in the dipterans *D. melanogaster* and *Chironomus* sp. these muscles are primed using, as a founder template, the remains of larval oblique muscles (LOMs), which are not degraded during metamorphosis, and to which FCM fuse [20, 45]. In late L4 instar of *A. aegypti* we observed, in dorso-coronal sections of the thoracic region, three structures per hemithorax corresponding to putative DLMs primordia (Fig. 2a, b, e, numbered 1, 2 and 3) and, in agreement to those expected by comparison with *D. melanogaster* and *Chironomus* sp., each of them have four myotubes, suggesting the splitting of the putative initial three LOMs (Fig. 2a, and cf. [46]). At the anterior end, putative DLM myotubes have notable membrane extensions forming the myotendinous junction (MTJ), connecting the muscle in formation with the epidermal tendon cells at the anterior thoracic epithelium (Fig. 2a–c, black arrowheads, and 2e, green arrows). In the attachment sites, terminal ends of myotubes have a heterochromatic zone with elongated nuclei (Fig. 2c) and MTJ are formed between anterior end of myotubes and thoracic epithelial cells (Fig. 2a–c, black arrowheads and 2e, green arrows). At this stage, myoblasts are observed among fibers (Fig. 2d). In an oblique section three fibers per hemithorax were observed, each formed by four fibers possibly in splitting process (Fig. 2e, red asterisks) corresponding closely to those observed in *D. melanogaster* and *Chironomus* sp. At the posterior end the primordial myotubes are suspended in the hemocoel and structures suggesting the start of MTJ formation are seen (Fig. 2e, green arrowheads). No variation in the number of primordial DLM myotubes were observed.

### Actin distribution in IFM primordia of L3 and L4 larval stages

**Fig. 3:**
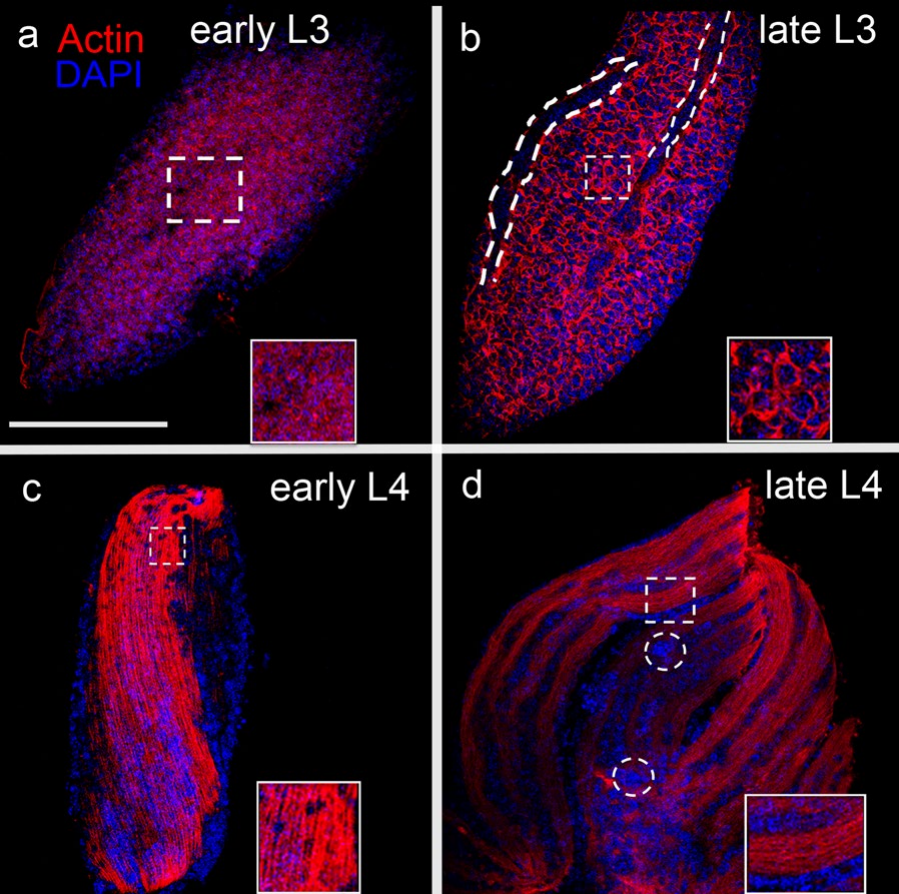
Actin assembly and organization in multinucleated primordial IFMs. **a**–**d** IFM primordia of early and late phases from L3 and L4 instars were stained with Rhodamine-Phalloidin (red) to highlight actin filaments and nuclei with DAPI to visualize nuclei (blue). **a** In early L3 primordium, disperse Phalloidin-labelled short actin filaments widely distributed were observed. **b** In late L3 instar, the muscle primordia showed abundant actin. At this stage primordia showed stripes forming sections-like along the primordium (dotted lines). **c** The early L4 primordium, actin polymerization led to the formation of immature myofibrils organized in bundles within myotubes. **d** The late L4 primordium showed a larger size and abundant immature myofibrils forming muscle fibers. At this stage, the nuclei were observed forming rows along the muscle fibers and, some of them showed a larger size than the others (dotted circles). Insets show enlargement of dashed boxes. Scale bar: 100 μm

To study the actin dynamics during myofibril formation, IFM primordia from L3 and L4 instars were carefully dissected sectioning the cell extensions that connect them to the epidermal attachment sites (Additional file 2: Fig. S2), and primordia were phalloidin-rhodamine stained to highlight F-actin, and DAPI stained to visualize nuclei. In early L3, short F-actin filaments were diffusely distributed in the primordia (Fig. 3a, inset); for late L3, the abundance of F-actin increased in size and quantity in myoblasts cytoplasm (Fig. 3b, inset). Besides that, on the surface of the primordium grooves short F-actin filaments are present, probably corresponding to the myotubes in formation (Fig. 3b, dotted lines). In early L4, as part of the myofibrillogenesis process, the actin forms filaments organized in bundles along the fiber in formation, in concordance with myotube orientation (Fig. 3c, inset). In late L4, actin filaments were organized in bundles within structures recognizable as premyofibrils, suggesting that the actin synthesis and companion proteins, necessary for myofibrillogenesis, is maybe taking place (Fig. 3d, inset). The nuclei in IFM primordia were organizing from late L3, where they are in individual myoblasts, through L4, where the nuclei are visualized in rows, corresponding to syncytial myotubes. In addition, there are big nuclei scattered between the nuclei rows (Fig. 3d, dotted circles).

### Distribution and organization of actin, myosin and tubulin in the L4 DVM primordium

**Fig. 4:**
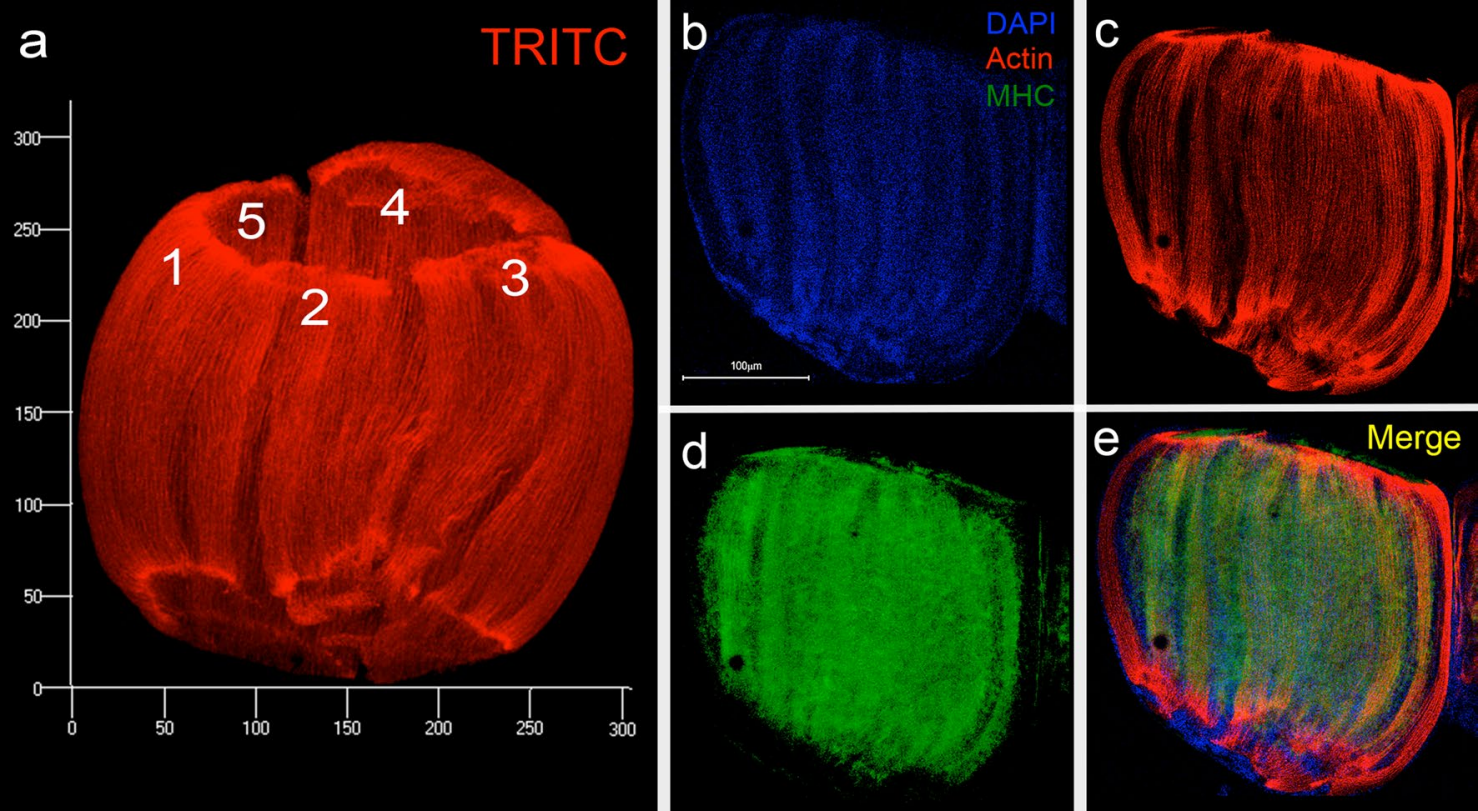
F-actin and myosin filaments patterns in DVM primordia. L4 DVM  primordia obtained from L4 instar was stained with TRITC-Phalloidin (red), DAPI (blue), and anti-Myosin Heavy Chain antibody (MHC) (green) to observe the F-actin and nuclei distribution in forming myofilaments. Images of optical slices were collected by confocal microscopy and a projection constructed using the ZEN program (Zeiss). **a** A structure showing hundreds of immature myofibrils forming five possible muscle fibers (1–5, clearly separated). The DVM primordium showed an approximate size of 300 μm in both length and width. **b** Nuclei forming rows within the primordium. **c** The same primordium showing hundreds immature myofibrils formed by F-actin whitin muscle fibers. Also, both ends of primordium were intensely stained with actin. **d** Myosin distribution in primordium shows distribution parallel to actin. **d** Merge. Scale bars: **b**–**e** 100 μm

Due to the importance for muscle fibers assembly, the presence and distribution of F-actin (Fig. 4a), myosin heavy chain (MHC) (Fig. 4b–e) and tubulin (Additional file 4: Fig. S4) were explored in *A. aegypti* IFM development using TRITC-P or phalloidin-rhodamine stain, anti-MHC and anti-tubulin antibodies and confocal microscopy. The F-actin distribution was studied in a late L4 primordial DVM using TRITC-P stain (Fig. 4a, c). The integration of multiple two-dimensional images at different depths allowed us the three-dimensional reconstruction of a DVM primordium of approximate size of both 300 × 300 μm length × wide with the F-actin, organized in five separated fibril bundles, containing hundreds of actin fibrils oriented along the major axis of the primordium (Fig. 4a).

Muscular myosin was very abundant (Fig. 4d), its distribution partially colocalize with those of F-actin (Fig. 4c–e), suggesting a premyofibrill pattern. At this stage the alignment of the nuclei along the primordium was already evident (Fig. 4b and Additional file 3: Fig. S3), in syncytial myofibers in formation. Tubulin staining distribution, as those of actin and myosin, render a fibrillar pattern along the primordium, indicating microtubules aligning along the fibers (Additional file 4: Fig. S4).

### Ultrastructural analysis of myoblast fusion during IFM formation

**Fig. 5:**
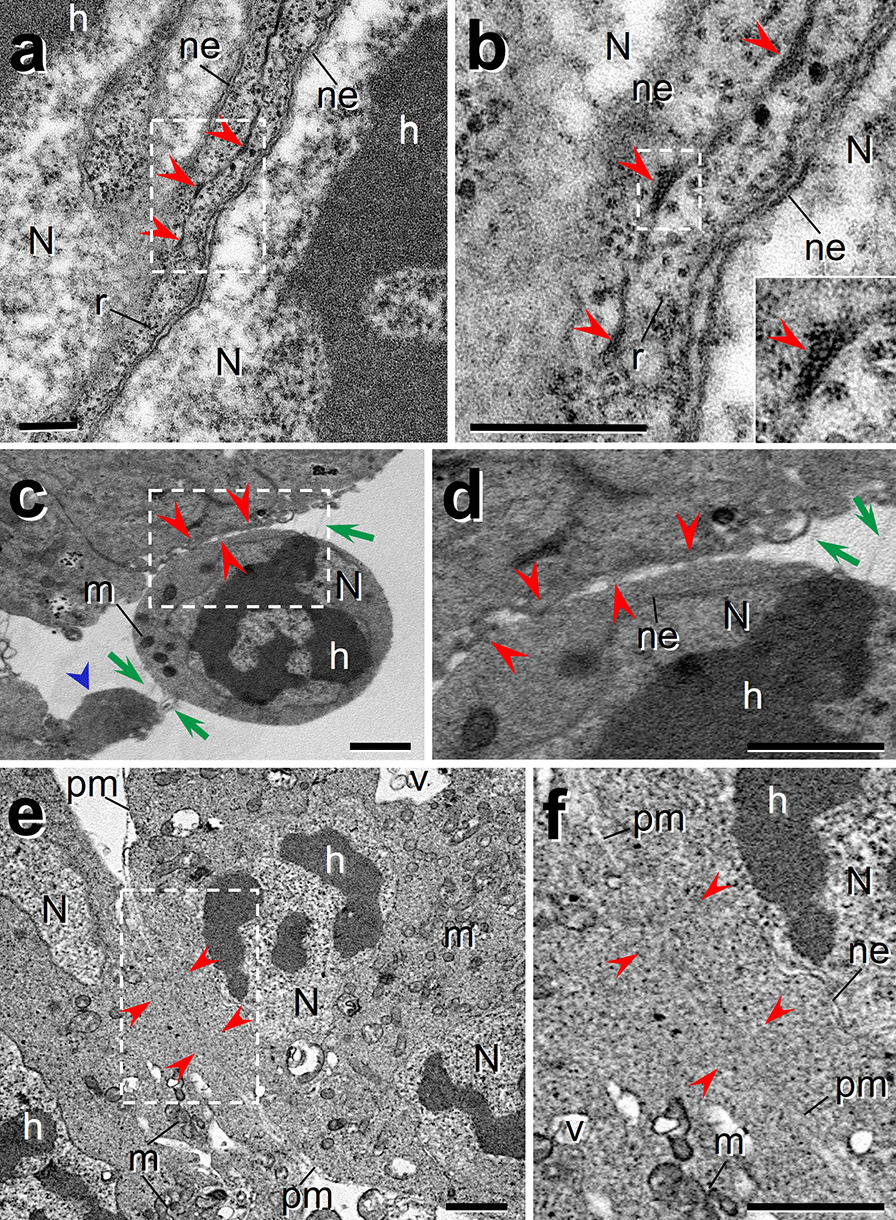
Myoblast fusion in IFM development. L4 primordia were dissected and analyzed by TEM to show details of cell structure and organization during IFM developing. **a**, **b** During early fusion steps prefusion vesicles are formed and coalesce (red arrowheads) close to the membranes apposition zone. **b** Magnification of white dotted box in a, where small vesicles were observed; inset, magnification of dotted box showing vesicles surrounded by electron-dense material. **c**, **d** A rounded myoblast showing membrane extensions such as filopodia-like (green arrows), another cell shows a podosome-like protrusion (blue arrowhead) and, at cell contact sites some fusion pore-like structures were also observed (red arrowheads). **d** Magnification of white dotted box shows detail of the filopodia-like structures (green arrows), and fusion pores, where some vesicles between the pores-like were seen (light areas between red arrowheads). **e**, **f** Fusing myoblasts membrane apposition zone where possible pores are forming and the cytoplasm is crossing from one cell to another are shown (red arrowheads). **f** Magnification of white dotted box in e, showing possible cytoplasmic continuity among two cells through two pore-like structures (red arrowheads). *N* nucleus, *ne* nuclear envelope, *h* heterochromatin, *m* mitochondria, *pm* plasma membrane, *r* ribosomes, *v* vesicles. Scale bars: **a**, **b** 0.5 µm; **c**–**f** 3 µm

In *D.* *melanogaster* it has been stablished that during early IFM formation, FCM recognize and adhere to FC in DVM, or to myotubes (larval scaffolds) in DLM, followed by cell fusion, forming syncytial myotubes. To study in *A.* *aegypti* FCM fusion to their targets, IFM primordia obtained from L4 were analyzed by TEM. We observed that during contact establishment between fusion myoblasts to FCM or myotubes, several electron-dense contact points were visualized (Fig. 5a, red arrowheads and dotted box), resembling closely the prefusion complexes identified in the *D. melanogaster* fusion process. In the contact points the vesicles appear paired between the apposed plasma membrane (Fig. 5b, red arrowheads and dotted box); in addition, electron-dense material associated with plasma membrane and surrounding the vesicles was also observed (Fig. 5b and insert, red arrowheads).

Other structures possibly related to cell fusion, resembling filopodia-like (Fig. 5c green arrows), and podosome-like (Fig. 5c blue arrowhead) protrusions between a myoblast (M) and a possible myotube (MT) were observed. Interestingly, fusion pore-like structures were also found at cell contact sites (Fig. 5c, d red arrowheads), where a possible exchange of cytoplasmic material is suggested. Besides, vesicles along apposed plasma membranes between fusion pores are formed (Fig. 5d, clear space among red arrowheads). In these regions with intimate contact between cells, cytoplasmic continuity could be observed between adjacent cells (Fig. 5e, f red arrowheads).

In development of muscle of *D. melanogaster*, multinucleated myofibrils are produced by myoblast fusion. We analyzed multiple cuts of IFM primordia of late L4 instar of *A. aegypti* mosquito and observed populations of presumptive individual membrane-bordered myoblasts (Additional file 5: Fig. S5a), others possibly continuing to the fusion process forming multinucleated rows (Additional file 5: Fig. S5b–d) and membranes bordering forming multinucleated myotubes (Additional file 5: Fig. S5d, green arrowheads). At these larval stage myoblasts nuclei are pleomorphic with relaxed chromatin with abundant granular material and in the cytoplasm there are many small mitochondria.

### Myofibrillogenesis during developing IFMs in the larval stage

**Fig. 6:**
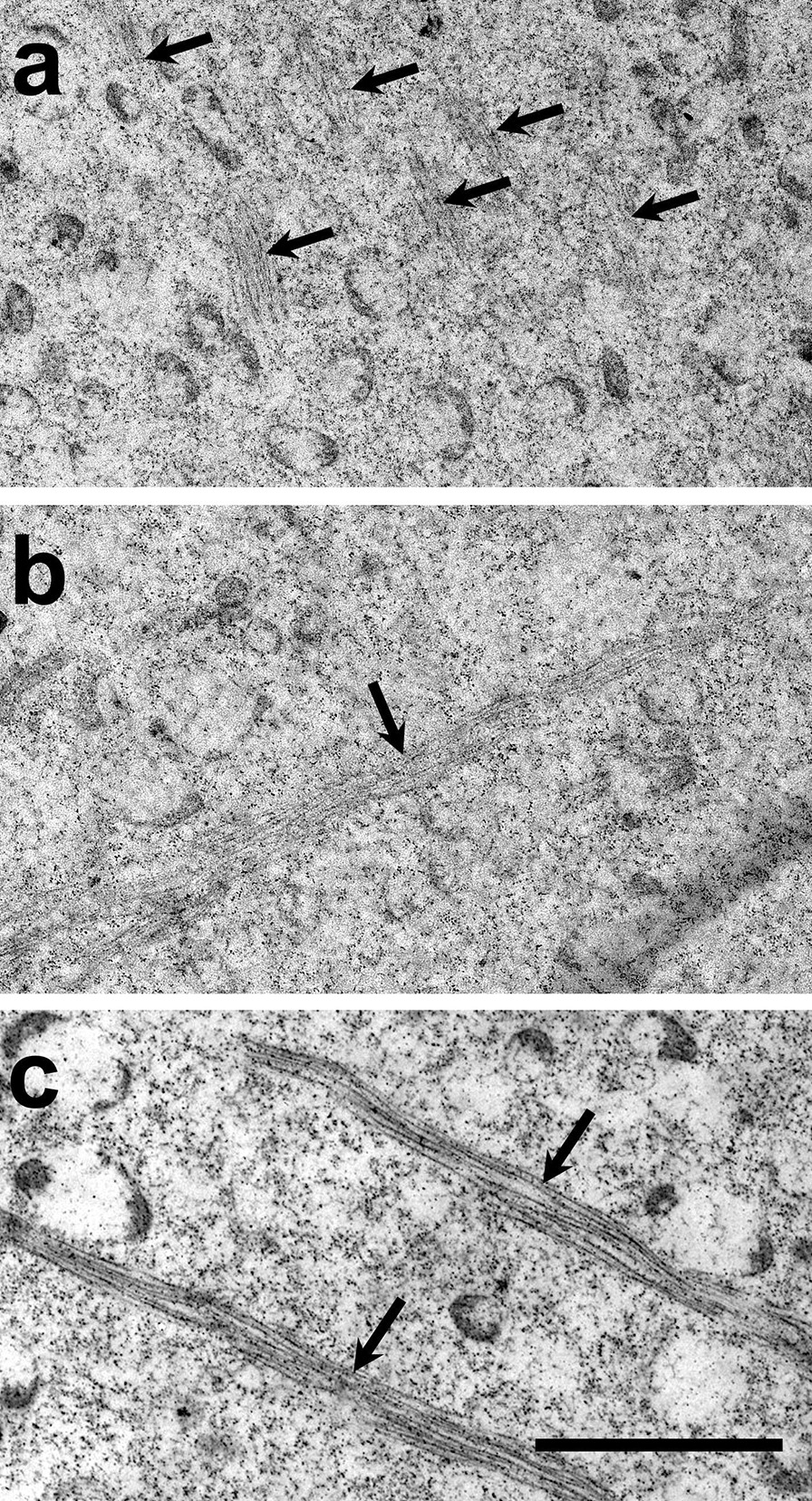
Actin organization during developing IFMs in L3 and L4 stages. Myofibrils formation during IFM development was analyzed by TEM. **a** In late L3 primordia, short actin filaments arrays (arrows) were observed. **b** Early L4 primordium showed long filaments. **c** Late L4 primordium showed greater number of long and dense fibers, possibly immature myofibrils. Scale bar: 2 µm

Previously, we observed F-actin distribution in L3 and L4 primordia of *A. aegypti* mosquito by confocal microscopy (Fig. 3). To analyze deeper the F-actin structure during IFM development, we conducted observations by TEM. In late L3 primordia, there are short actin filaments arrays (Fig. 6a, black arrows) which, in early L4 evolve to longer and thicker filaments, corresponding to those observed in premyofibrils (Fig. 6b, black arrow). Subsequently, in late L4 primordia immature myofibrils with thicker and dense F-actin were observed forming very long filaments (Fig. 6c, arrows). These results agree with those shown before.

### Myofibrils and sarcomere maturation during pupae and adult stages

**Fig. 7:**
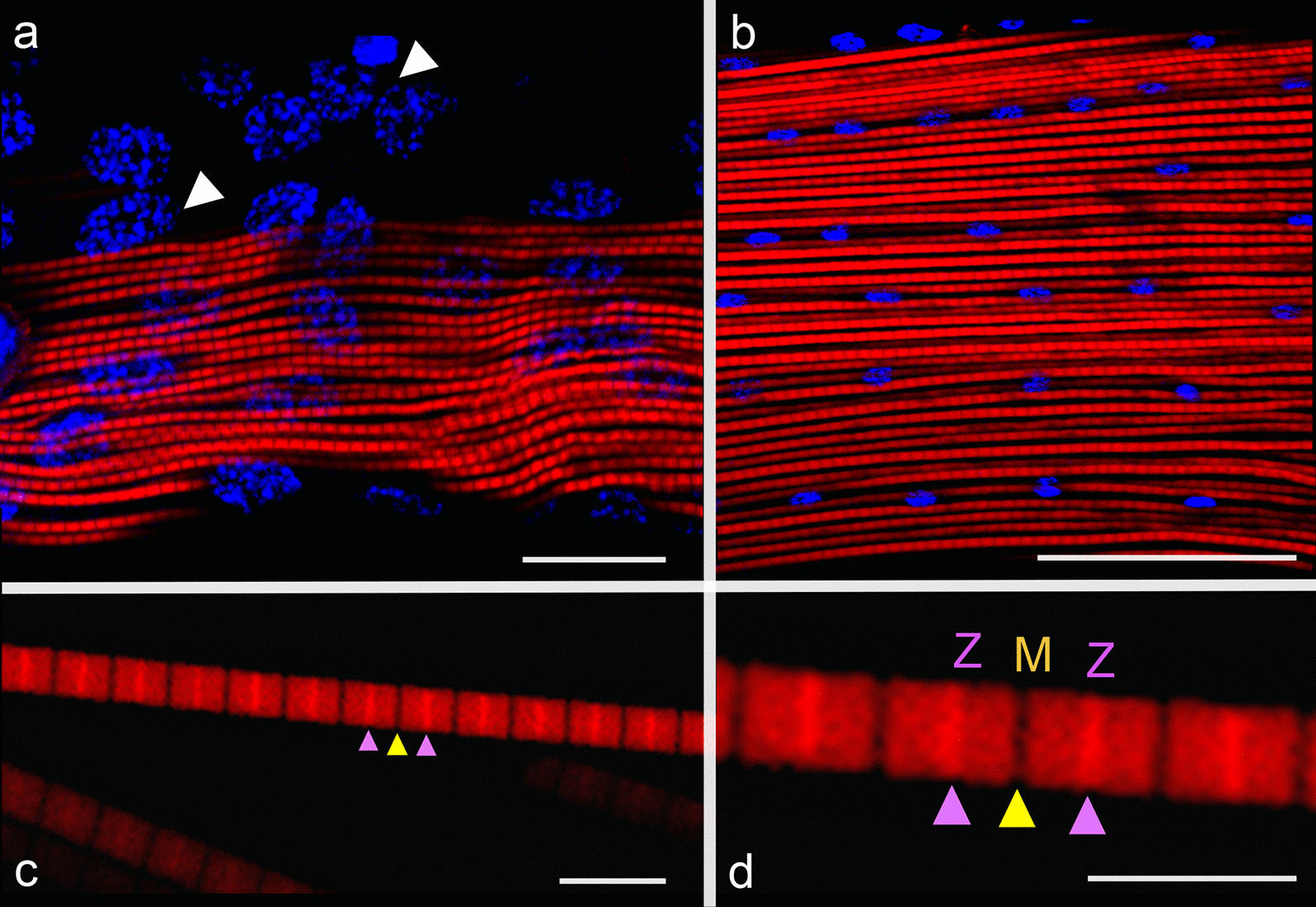
Myofibrils and sarcomere maturation during pupae and adult stage. The maturation process of myofibrils and sarcomeres was analyzed by confocal microscopy, staining F-actin with rhodamine-phalloidin (red) and nuclei with DAPI (blue). **a** In late pupal stage, the myofibrils had a syncytial structure, with nuclei showing a relaxed chromatin organization (white arrows). **b** In adults, uniform mature myofibrils containing long and organized mature sarcomeres were found. In both pupae and adult IFMs, the nuclei forming rows were observed. **c** The isolated myofibrils showing the classical structure of mature sarcomere, including the bright Z disc and the dark M-line (magenta and yellow arrowheads). **d** Magnified image of sarcomere structure. Scale bars: **a**–**c** 50 µm; **d** 10 µm

The myofibril F-actin assembly in *A. aegypti* IFMs was studied in the pupal and adult stages by confocal microscopy. We observed that during early pupal stage the myotubes formed during larval stages continued to growth converting into myofibers. The muscle fibers from late pupal DLMs were dissected and analyzed, showing large, thick and wavy muscle fibers (Fig. 7a), but in spite the F-actin is structured forming short segments, sarcomeres are immature yet. Furthermore, nuclei have chromatin loosely packed suggesting high transcriptional activity (Fig. 7a, white arrowheads). Upon the adult stage, muscle fibers grew to fill the entire thorax, corresponding this phase to hypertrophic growth and assembly of myofilaments to form myofibrils and finally, the differentiation of sarcomeres. The analysis of adult DLMs three day after emergence showed totally structured myofibers formed by hundreds of uniform mature myofibrils with long and organized mature sarcomeres, furthermore the nuclei are smaller and arranged in rows parallel to myofibrils (Fig. 7b). Focusing on adult muscles, the mature myofibril components, the sarcomeres, were clearly observed, with characteristic dark M-lines and the bright Z-discs (Fig. 7c, d, yellow and magenta arrowheads, respectively).

### Ultrastructure of IFM sarcomere in pupae and adult stages

**Fig. 8:**
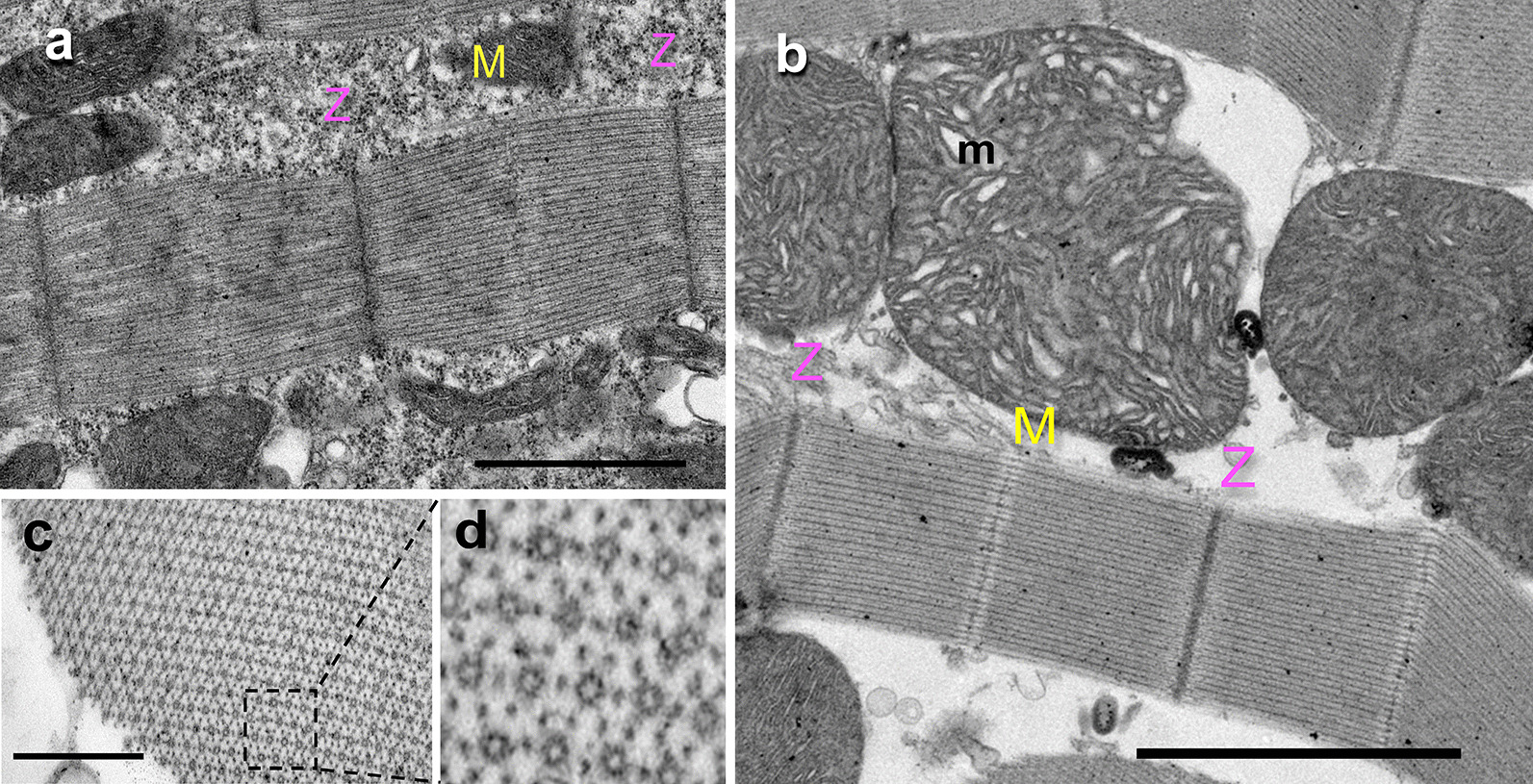
Sarcomere ultrastructure in pupae and adult IFMs. IFMs from *A. aegypti* pupae and adult were dissected and analyzed by TEM. **a** In longitudinal sections of pupae muscle, still in growth, short sarcomeres well-structured (~ 1.95 μm), showing the Z-discs and M-lines (magenta and yellow colors, respectively) were observed. Large and elongated mitochondria were seen. **b** Adult mosquito muscles showed sarcomeres completely formed (~ 2.90 μm), with clearly defined Z-discs and M-lines. Numerous giant mitochondria with tightly compressed cristae were found alongside the flight muscles. **c** Cross section of a mature myofibril showing the sarcomeres with hexagonal arrangement. **d** Magnified image of the dashed box in **c**, showing thick myofilaments (myosin, visualized as large hollow circles), each surrounded by six thin myofilaments (actin, visualized as small black spots surrounding each thick filament), in a relation of 6:1. Scale bars: **a** 1 µm; **b** 2 µm; **c** 0.5 µm

IFMs dissected from late pupae and adults were analyzed by TEM. In late pupae stage, the immature myofibrils and sarcomeres are already assembled; pupal sarcomere showed ~ 1.95 μm in length and Z-discs and M-lines are already well defined (Fig. 8a); mitochondria had a typical elongated shape, small size and tubular cristae. In adult mosquito, the myofibrils were structured, showing typical sarcomeres bordered by two Z-discs and M-line, which reach ~ 2.90 μm in length (Fig. 8b). At this stage, mitochondria reached a giant size, with polymorphic cristae, suggesting mitochondrial fusion. Finally, in cross sections of a mature myofibril the sarcomeres showed the classical hexagonal lattice structure (Fig. 8c, d dotted box expanded), where thick myofilaments aligned regularly with a uniform diameter, and visualized as large hollow circles surrounded by six small black spots, corresponding to thin myofilaments of actin (Fig. 8d).

## Discussion

For mosquitoes, flying is fundamental for survival, population dispersion and disease transmission, and there are vector control strategies based on interfering with mosquito muscle formation with significant effects on disease transmission [47–49]. Notwithstanding, despite the importance of mosquitoes as vectors of infectious diseases, which are major health concerns, the knowledge of the development of the IFMs, that give the potency for wing beating [50, 51] is limited. In contrast, in *D. melanogaster* there are numerous morphologic and molecular biology studies about IFM development, constituting a wide study field with numerous tools to study it, including mutants, antibodies and manipulation methods [45]; these tools are necessary to develop for mosquito studies but the knowledge about fly muscle development was very helpful for our study.

In this work we present an initial description of *A. aegypti* IFM development. It is important to mention that we found notorious differences in the development of *A. aegypti*, belonging to suborder Nematoceran (a “lower” dipteran), with those of *D. melanogaster*, a Brachyceran (a “higher” dipteran), and we found interesting similarities to *Chironomus* sp., which is another lower dipteran Nematoceran, including the timing of the muscle development [52–54]. In *D. melanogaster*, as in all higher dipterans, there are three larval stages, followed by a puparation, with a prepupal stage and, after, during the metamorphosis (pupation) most larval muscles are dissolved, and formation of adult muscles, including IFMs is accomplished. In comparison, in *A. aegypti* and *Chironomus* sp., there are four larval stages and the IFM development begins actively during the last two and proceed during pupation. We were able to identify in *A. aegypti*, as early as L3, clusters of cells that will form the IFMs.

In dipterans there are two IFM groups of fibrillar muscles, with major differences during development: the DLM constructed priming on larval oblique muscles (LOMs), which persist after metamorphosis and are used as templates for myoblasts migration and fusion to form the adult muscle; and DVM, formed de novo by migration of myoblasts to specific locations where they conglomerate around founder cells [55, 56]. Here, we show that at the front of L4 *A. aegypti* thorax, DLM are formed of three fascicles per hemithorax, attached to frontal thorax at different levels, with a structure similar to LOMs to which myoblast-like migrate and fuse, then these muscles possibly split to originate six myotubes per hemithorax, similarly to those happening in *D. melanogaster* and *Chironomus *sp. [20, 45, 52–55] and, this fascicle conformation coincides with images presented before in *A. aegypti* (cf. [46]). In comparison, the DVMs are constructed de novo, and our images strongly suggest the migration of FC and FCM, which have been proposed that originate from wing imaginal disk cells [20], forming primordia [52, 53], which will evolve to form myotubes, myofibers and sarcomeres. Interestingly, we observed that the putative IFM primordia of *A. aegypti*, where primordial myotubes are contained, were enveloped with a basement membrane, as has been observed in *Chironomus* sp. [52, 53]. When the IFM precursors and adult muscles are compared between *D. melanogaster* and *A. aegypti*, major differences are found, including the structure of primordia and the final number of fibers in the adult (Additional file 1: Fig. S1 and Table 1) [10, 20, 57–61]. In a previous report, the number of adult IFM fibers in *A. aegypti*, was compared among a population of an inbreed laboratory strain with three field collected populations, and significant differences in the number was observed, and differences were more frequent in DVMs of laboratory strains. Furthermore, the authors detected DLM splitting of muscles leading to side number asymmetry and formation of ramified adult muscles, which probably affects the ability to fly. The higher variation in laboratory strains is explained by arguing that in nature the modified individuals are eliminated in a “stabilizing selection” [10]. These results coincide with our observations where the number of DLM fascicles was constant, but in DVMs in the groups 1 and 2, and despite the small number of samples analyzed, variations in myotube numbers were seen [56]. The regulation of muscle size and number during IFMs formation in *D. melanogaster* is controlled by a balance between fusion and proliferation during larval and pupal stages [56, 62–64].

**Table1:** Indirect flight muscles and tergal depressor of the trochanter muscle in formation in adults of *Drosophila melanogaster* and *Aedes aegypti*

*Drosophila melanogaster*^a^				*Aedes aegypti*		
Group	Fascicles in Pupa		Adult muscles	Group	Fibers in L4^j^	Adult muscles^c,d^
*DVM (per hemithorax)*						
I	3		3	I	4	4
II	2		2	II	5	5
III	2		2	III	4	4
Total muscles			7			13^c^^,j^

*Drosophila melanogaster*^b^				*Aedes aegypti*		
		Fibers	Adult muscles		Fascicles in L4^j^	Adult muscles
*DLM (per hemithorax)*						
		6	12		3	12^c^^,h^

*Drosophila melanogastere*^e−g^				*Aedes aegypti*		
			Adult muscles		Fascicles in L4	Adult Muscles^h,i^
*TDT (per hemithorax)*						
			1 (~ 32)		ND	1

^a^Fernandes et al. [20]; ^b^Fernandes et al. [56]; ^c^Beckett and Townson [10]; ^d^Beckett and Macdonald [57]; ^e^Peckham et al. [58]; ^f^Eldred et al. [59]; ^g^Swank [60]; ^h^Jobling [44]; ^i^Becket [61];^ j^This work

In this work, in *A. aegypti* larvae we recognized three groups of cells (primordia) at DVM locations as early as L3, which we followed through L4 and pupa up to generate the adult muscles by hypertrophic growing. Analyzing the DVM development in *A. aegypti* we observed cells with locations and “teardrop” and spindle morphologies, corresponding to *D. melanogaster* FCM which migrate from the wing imaginal disc to specific sites where primordia will be developed forming nascent fibers. Our observations of FCM-like cells in *A. aegypti* DVM primordia allow us to reasonably propose, that in this mosquito FCM cells go through a similar process with active division, migration and location out and inside the primordial myotubes [62]. In addition, in late L3 and early L4 the FCM associate to founder cells, which have distinguishable big and heterochromatic nuclei at the center of nascent myotubes, and, as result of cell fusion, syncytial myotubes are formed.

Another kind of putative primordium was observed in the expected location, in respect *D. melanogaster* and *Chironomus* sp., for the TDT muscle and, as it was expected, it has a different organization, without myotube divisions, corresponding to a tubular muscle. Tubular muscles have major differences in respect to DVM, as we observed here, including the radial distribution of nuclei and FCM fused in a single syncytial compartment and a variable number of fibrils (Table 1) [65–67].

In *D. melanogaster*, it has been reported that mechanical tension during the muscle formation depend on an attachment process, via myotendinous junction (MTJ) formation, which is necessary to stabilizing the myofibrils during assembly, and become innervated by motoneurons through neuromuscular junctions to mature into stable and functional muscles [63, 68–70]. In *A. aegypti* we observed at L4 three DLM fascicles per hemithorax bound to the anterior epithelial wall of the thorax. At this stage, siphons are visible, DLM are attached to the epithelium only at the front of the thorax, close to the respiratory trumpets by mean of elongated extensions of tendon cells. In this study we did not observed the fixation of DLM to the posterior thorax lamina, which in *D. melanogaster* is formed by tendon attachments that are early stablished at both muscle sides, maintaining tension during development [68–70]. In addition, referring to the mechanism of MTJ formation in *A. aegypti*, we observed that at posterior DLM end, there are cells with enlarged nuclei, like those that contact with thoracic epithelial tendon cells at anterior end, besides possible MTJ in formation (Fig. 2e, green arrowheads). Furthermore, at L4 primordia of both DLM and DVM, were observed actin and tubulin rich plates at both ends of muscle fibers (data non shown), which could be related to the construction of MTJ, as it has been reported for *D. melanogaster* [68–70]. Many issues are open in *A. aegypti* in respect to MTJ and neuromuscular junctions in formation as the identification of integrins and other extracellular matrix molecules participation [39, 63–66, 68–73].

At L4, the three DLM have four myotubes and this number was constant and is in accordance with images previously reported (Fig. 2 and cf. [46]). In contrast, in DVM we observed that the numbers of myotubes for DVM 1, 2 and 3 are majorly 4, 5 and 4, respectively, but some variation was observed, in agreement with previous authors and it is difficult to explain [10].

Interestingly, DLM location in L4 coincide with the site of expression of reporter genes under the control of Aeact-4 promoter, a female-specific promoter used for transgenic mosquito construction of *flightless* phenotype in females of *Aedes* spp. for vector control [48, 49], suggesting that this promoter rules expression specifically in the DLM. In addition, the expression ruled by Act88F from *D. melanogaster,* when cloned in the *Culex quinquefasciatus* mosquito*,* directed the expression of a reporter in all the IFMs [74], and the *D. melanogaster* promoter for Act79B gene directs the expression specifically in TDT in the fly [65], indicating the diversity and specificity of the promoters involved in IFM development in dipterans, demonstrating that there are general and specific promoters for IFM and other thoracic muscles and, research of this issue will be important for designing new constructions aimed to vector control.

During *A. aegypti* L3 instar, myoblasts migrate to form small clusters located at defined places, priming the construction of DVM. Inside the nascent fascicles, founder cells (FC) define the myotube formation recruiting fusion competent cells (FCM), which divide actively, and it is possible to recognize their presence by their characteristic morphologies, that are considered hallmarks of this kind of fusing cells [62]. Myoblast fusion process involve the formation of filopodia and podosome for the initial contact and the organization of prefusion vesicles that coalesce around points where the integration of myoblasts to the myotubes are happening [30, 32–34, 74], generating syncytial structures. Here we present in *A. aegypti* images similar to those of fusion events during *D. melanogaster* myotubes formation and concomitant to this, prefusion vesicles which could conduct to the formation of syncytial-fused tubes with aligned nuclei forming rows in the muscle axis during early formation of contractile adult muscle [32–34, 54, 62, 75].

For the organization of muscle components during development actin, myosin and tubulin are fundamental for sarcomere construction [39–42, 76, 77]. General organization of F-actin and myosin was studied during the *A. aegypti* IFM myogenesis. F-actin abundance increase from L3 to pupa and organization evolved from short structures in early L3 muscle primordia, in the cells that are not yet fused. After, in late L3, when the cells fusion is in progress, F-actin was structured in short filaments, and at L4 F-actin fibers are longer and along the myotubes. Simultaneously, in L4, muscular myosin was strongly produced and associated to actin filaments, forming premyofibrils. Furthermore, in *A. aegypti* IFM L4 primordia we observed dense longitudinal microtubules arrays. During IFM developing of *D. melanogaster* a microtubules array, with their associated proteins, as well as non-muscular myosin are also involved in nuclei arrangements forming rows in the syncytial myofibers, elongation and muscle shaping [20, 21, 39, 76–81]. At pupal stage, myosin and actin organize in premyofibrils with immature sarcomeres then, hypertrophic muscle growth happens, accompanied by mitochondria extensive growth and fusion [82, 83]. IFMs have a very high-energy requirements which are supplied by specialized mitochondria [50, 51, 82, 83]. Here we show that *A. aegypti* flight muscles undergo major changes in their structure in the pupa to teneral transition. Mitochondria proliferate in pupa and coalesce rendering giant mitochondria with tubular cristae during final steps of myogenesis up to the adult stage. In *D. melanogaster* IFM mitochondria has been described the genetic control of outer and inner membranes fusion [82, 83], and the identification of these genes is an interesting research perspective in *A. aegypti*. On the other hand, teneral mosquito’s sarcomere evolve to attain, in the adult, the definitive organized pseudocrystal myosin: actin structure, in a 1:6 relation, similar to those observed in the fly [42, 84, 85]. In *D. melanogaster*, the muscle growth and myofibrillogenesis are activated by several transcription factors, as retinoblastoma tumor suppressor (pRB), which activates the transcription of myogenic genes in an E2F-depending manner [86, 87]. Other cardinal topic to investigate for IFM development in the mosquito, is the myoblast fusion process, for which, in *D. melanogaster*, have been described many genes which are orderly expressed and can be used to label stages and activities during the process, and their products interacts very specifically [88]. These and other issues are unknown in *A. aegypti* and they should be investigated, increasing the observations between developmental stages, in order to discover developmental mechanisms for this and other mosquitoes, and to propose new options for mosquito control.

## Conclusions

In this work we present an initial description of the IFM myogenesis of the *A. aegypti* mosquito and compare the process with those described for *Chironomus* sp. and *D. melanogaster*, the most advanced dipteran model. In this work the fly myogenesis knowledge was very useful as an important outline, that allowed us to identify cells and structures, but major differences are pointed, and it is clear that many mechanisms and molecules when studied in the mosquito, will present interesting variations. In addition, although many hypotheses can be advanced for the IFM myogenesis in *A. aegypti*, based on mechanisms described for the fly, many challenges must be overcome to test them: it is difficult the direct visualization of migrating cells, there are limited methods to study the dynamics of forming structures and a complete mutants and antibodies collections should be build up. For the future, based on the extraordinary knowledge of *D. melanogaster* development, we will be able to do “mining” in the genomic resources accessible for *A. aegypti* to identify development relevant genes, predict the presence of specific proteins and produce recombinant molecules for antibody production to detect the *D. melanogaster* homologs and their expression.

## Methods

### Mosquitoes

*A. aegypti* mosquitoes (Rockefeller strain) were obtained from the Instituto Nacional de Salud Pública, Cuernavaca, Morelos, México. Mosquitoes were reared under insectary conditions (12/12 h photoperiod at 27 °C, 80% relative humidity and fed on 10% sucrose solution). Larvae were maintained in tap water and fed ad libitum with grounded cat pellets (Whiskas, Querétaro, México), previously sterilized. Larvae of different stages and pupae were separated into groups for better growth control. Female mosquitoes were mouse blood fed when necessary to maintain the mosquito colony.

### Muscles preparation

Muscles dissection was performed in *A. aegypti* as described for *D. melanogaster* [20]. Briefly, early and late stages of third (L3) (absence or siphon ejection, respectively) and 4th (L4) instar larvae and, early and late pupae (light and dark colored, respectively) were placed on a slide with a drop of cold 0.1 M sodium phosphate buffer (PBS) pH, 7.2. The mosquito head and thorax were excised from the main body with the help of dissection needles, cutting between the second and third abdominal segment. The thoraces were cut on the ventral side, the organs were discarded, and the opened thoraxes gently washed with cold PBS to remove the fat. The primordial muscles were recovered and, maintained in PBS on ice.

### Histological processing

Samples were processed for histological analysis as reported [89]. Briefly, whole larvae were fixed in Bouin’s solution (5% acetic acid, 9% formaldehyde, 0.9% picric acid) (Sigma-Aldrich). Samples were kept at room temperature (RT) for 24 h and transferred to 70% ethanol until paraffin embedding and then, the samples were dehydrated in progressive ethanol concentrations, infiltrated in Paraplast X-TRA (Oxford, St. Louis, MO) at 58 °C overnight, and embedded in Paraplast X-TRA using Simport histological cassettes (Quebec, Canada). Paraffin serial sections of 6 µm thickness were made and mounted in glass slides precoated with 3% 3-aminopropyltriethoxysilane (Sigma, St. Louis, MO.) in acetone. Sections were stained with aqueous Harris hematoxylin and eosin (HE) (Harris 1900) and observed under a Nikon microscope (Eclipse 600, Tokyo, Japan) using bright field optics.

### Sample preparation for confocal microscopy

For confocal microscopy, mosquito muscle primordia were prepared as reported for *D. melanogaster* tissues [90], with some modifications. Briefly, dissected *A. aegypti* primordial muscles were fixed with 4% paraformaldehyde and gently washed twice in PBS, permeabilized with PBS-T (PBS and 0.1% Triton) at RT for 2 h with gently shaking, and then soaked in blocking solution (PBS, pH 7.2, 10% FCS, 3% BSA and 10 mM glycine) for 30 min at RT; and washed twice in PBS for 20 min. To label F-actin, samples were treated with rhodamine-phalloidin (R415, Thermo Fisher Scientific) or Tetramethylrhodamine-Phalloidin (TRITC-P) (P1951, Sigma-Aldrich) in a 1:50 dilution in PBS at RT for 1 h in the dark. Samples were centrifuged 30 s at 1000 rpm and washed twice by gentle shaking in PBS for 20 min. Myosin labelling was performed using an Anti-Myosin Heavy Chain antibody (05-716, Millipore, USA) and the secondary antibody Goat Anti-Mouse Alexa Fluor 594 (ab150120). To label Tubulin, the samples were incubated with the anti-tubulin (ab44928, Cambridge, UK) and incubated with the secondary antibodies Goat Anti-Mouse Alexa Fluor 594 (ab 150120, Cambridge, UK). Samples were placed on glass slides and covered with 2 μl of Vectashield-DAPI for nuclei staining (Vectashield H-1200, dil. 1:400; Vector Laboratories, Inc., Burlingame, CA). The slides were observed under a confocal Zeiss L 700 microscope and images were analyzed using the ZEN lite Software (Zeiss, Jena, Germany).

### Transmission electron microscopy (TEM)

Primordia fixed in 2.5% glutaraldehyde and post-fixed with 1% osmium tetroxide in sodium cacodylate buffer for 60 min, were dehydrated in increasing ethanol concentrations and propylene oxide. The samples were embedded in Polybed epoxy resin (Polysciences, Inc. Warrington, UK) and polymerized at 60 °C for 24 h. Thin sections (60 nm) were obtained and contrasted with uranyl acetate and lead citrate. These sections were observed with a JEOL JEM-1011 transmission electron microscope [91, 92].

## Supplementary Information

**Additional file 1: Fig. S1.** DVM precursors from of *A. aegypti* larvae. **a**, **b** Coronal sections of late L4 thorax HE stained showed three transversally cut fascicles each side (right and left, labeled as 1, 2 and 3), corresponding to DVM I, II and III in both hemisegments. **a** Frequently fascicles composed of 4, 5 and 4 myotubes (numbered 1, 2 and 3, respectively) are observed on both sides. **b** Variability in DVM fasciculation was found in coronal sections of thorax showing on left side 4, 5 and 4 fascicles for DVM I, II and III, respectively. On right side, 5, 6 and 4 fascicles are observed for DVM I, II and III, respectively (dotted line circles). Red arrows point toward cephalic side of the larva. ca, cardia; gc, gastric caeca; fb, fat body. Scale bar: **a**, **b** 50 μm.

**Additional file 2: Fig. S2.** Morphology of the thorax of the L4 stage of *A. aegypti*. **a** Representation of the thoracic region where the position and location of the IFM primordia are marked. DLMs, black cylinders, cephalic to caudal oriented. DVMs, dotted green circles. **b** Light micrograph showing the location of the IFMs into the thorax of L4 instar. DLM primordia (red arrowheads) are attached by the anterior end of the frontal thoracic lamina, and DVM primordia are attached to the dorsal thoracic lamina (green arrowheads). Larval siphons are showed at anterior side (black arrowheads).

**Additional file 3: Fig. S3.** Actin organization and distribution of nuclei in L4 IFM primordium. IFM primordia from L4 were stained with Rhodamine phalloidin and DAPI and representative optical slices are presented. **a** The nuclei form long rows between the actin filaments, that are along the primordium forming premyofibrils. **b**–**d** Upper view of L4 primordium showing five fascicles with premyofibrils. **b** Nuclei DAPI stained; **c** filamentous actin labeled with rhodamine phalloidin. **d** Merge. Scale bar, 100 μm.

**Additional file 4: Fig. S4.** Tubulin organization in L4 IFM primordia. Developing isolated primordia from L4 *A. aegypti* larvae were stained using a specific anti-tubulin antibody and rhodamine-phalloidin to label F-actin. **a**–**d** A representative optical slice is presented. **a** Nuclei forming rows. **b** Actin filaments along the myofibrils. **c** Muscle primordia showed a tubulin distribution pattern parallel to actin filaments. **d** Merge. Scale bar 50 μm.

**Additional file 5: Fig. S5.** Morphology of individual myoblasts and multinucleated myotubes. IFM primordia from late L4 instar were dissected and analyzed by TEM. **a** Individual myoblast. **b**–**d** Fused myoblasts forming multinucleated myotubes with nuclei aligned in rows and bordered by membranes (green arrowheads). In d, two multinucleated myotubes closely associated, lined by membranes were observed. All nuclei are pleomorphic with relaxed chromatin and abundant granular material. Many small mitochondria are present. MB, myoblast; MT, myotube; N, nucleus; h, heterochromatin; ne, nuclear envelope; cm, cell membrane; m, mitochondria; v, vesicles; ve, vesicle with electron-dense material. Scale bars: **a** 1 μm; **b** 2 μm; **c**, **d** 5 μm.

## Data Availability

All data generated or analyzed during this study are included in this published article.

## Abbreviations

IFMs: Indirect flight muscles
TRITC: Tetramethylrhodamine B isotiocyanate
DLM: Dorsal-longitudinal muscles
DVM: Dorsal–ventral muscles
AMP: Adult muscle precursor cells
FCs: Founder cells
FCM: Fusion-competent myoblasts
LOM: Longitudinal oblique muscles
TDT: Tergotrochanteral muscle, jump muscle
HE: Hematoxylin–eosin
HC: Histochemical
ed: Epithelium of wing imaginal disc
fb: Fat body
L3: Larvae instar 3
L4: Larvae instar 4
MTJ: Myotendinous junction
DAPI: (4′,6-Diamidino-2-fenilindol)
MHC: Myosin heavy chain
TRITC-P: TRITC-phalloidin
TEM: Transmission electron microscopy
pRB: Retinoblastoma tumor suppressor
RT: Room temperature
PBS-T: Phosphate buffered saline-Tween 20
