# Supplementary figures and images for: Development of the indirect flight muscles of Aedes aegypti, a main arbovirus vector
Source: BMC Dev Biol. 2021 Aug 26;21:11. doi: 10.1186/s12861-021-00242-8 (PMC8394598; doi:10.1186/s12861-021-00242-8)

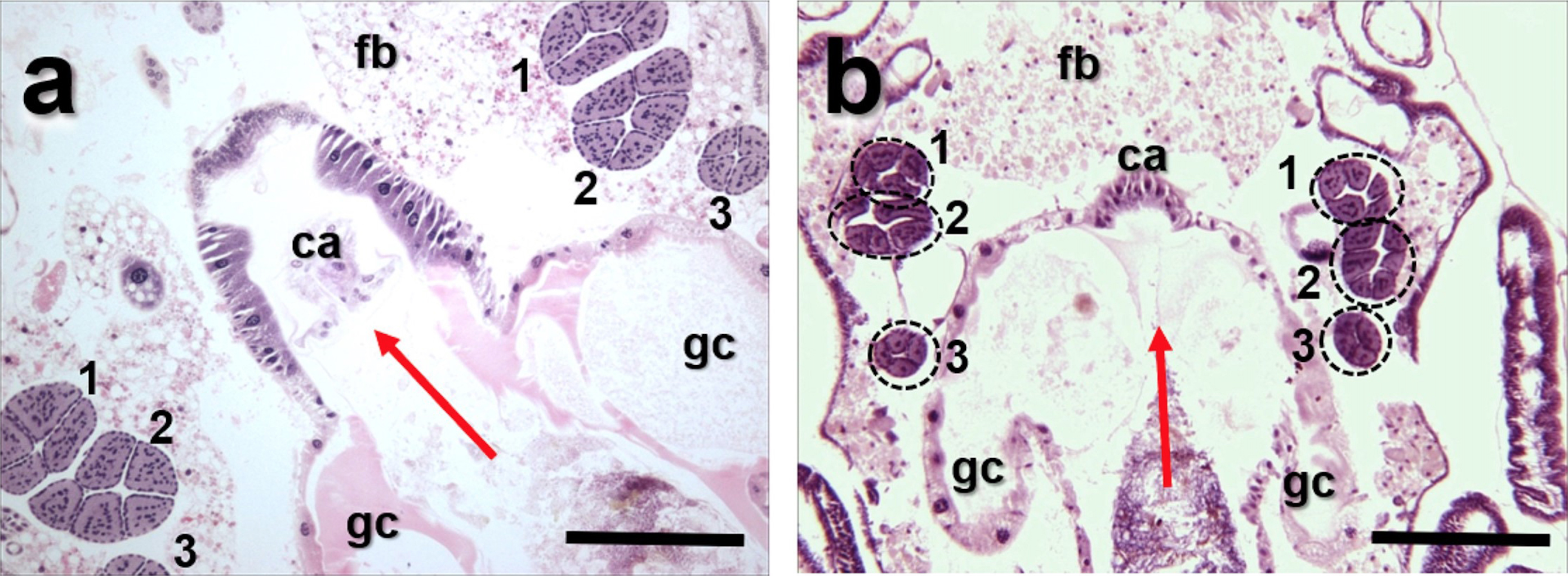

Supplement: Supplementary file 1 — Additional file 1: Fig. S1. DVM precursors from of A. aegypti larvae. a, b Coronal sections of late L4 thorax HE stained showed three transversally cut fascicles each side (right and left, labeled as 1, 2 and 3), corresponding to DVM I, II and III in both hemisegments. a Frequently fascicles composed of 4, 5 and 4 myotubes (numbered 1, 2 and 3, respectively) are observed on both sides. b Variability in DVM fasciculation was found in coronal sections of thorax showing on left side 4, 5 and 4 fascicles for DVM I, II and III, respectively. On right side, 5, 6 and 4 fascicles are observed for DVM I, II and III, respectively (dotted line circles). Red arrows point toward cephalic side of the larva. ca, cardia; gc, gastric caeca; fb, fat body. Scale bar: a, b 50 μm. [file 12861_2021_242_MOESM1_ESM.jpg]

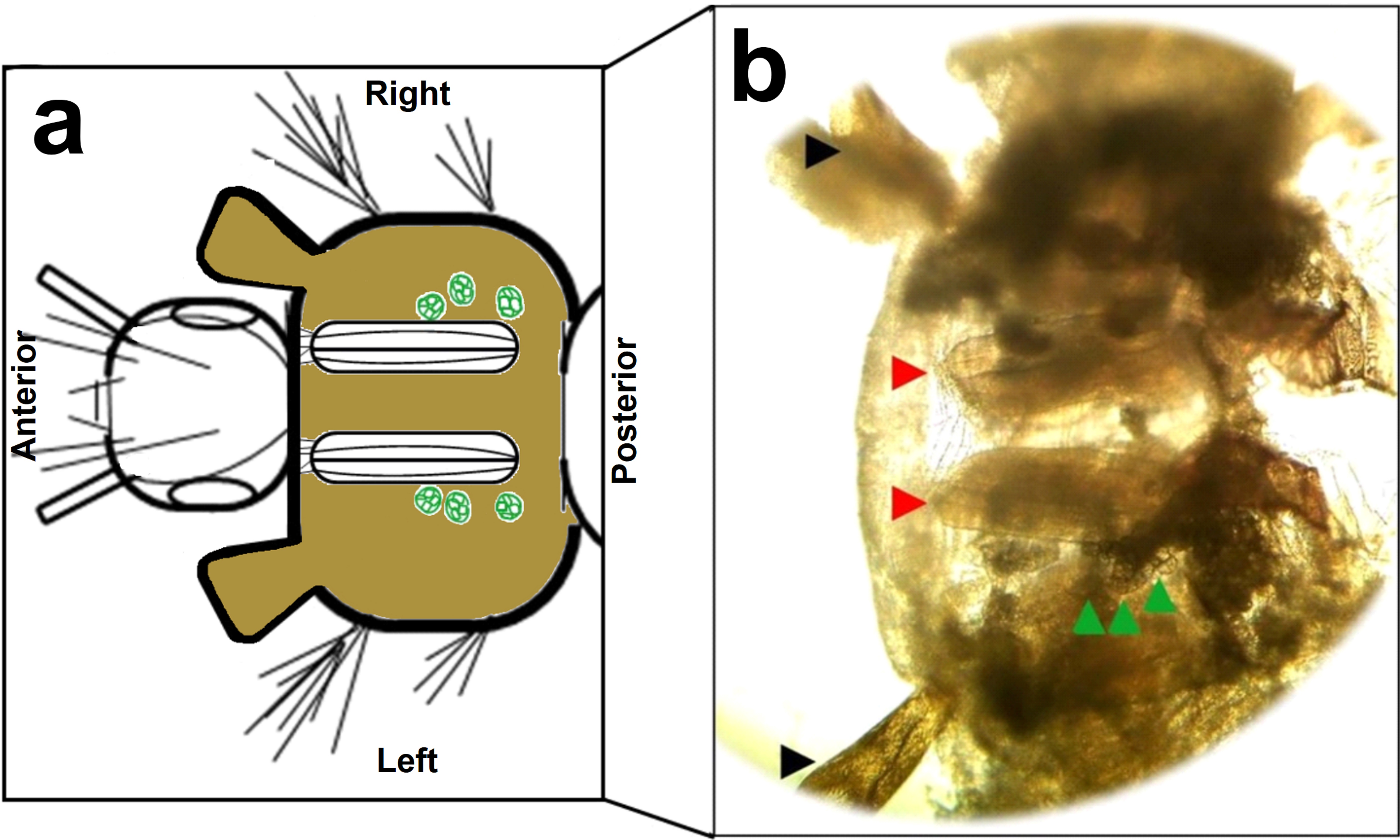

Supplement: Supplementary file 2 — Additional file 2: Fig. S2. Morphology of the thorax of the L4 stage of A. aegypti. a Representation of the thoracic region where the position and location of the IFM primordia are marked. DLMs, black cylinders, cephalic to caudal oriented. DVMs, dotted green circles. b Light micrograph showing the location of the IFMs into the thorax of L4 instar. DLM primordia (red arrowheads) are attached by the anterior end of the frontal thoracic lamina, and DVM primordia are attached to the dorsal thoracic lamina (green arrowheads). Larval siphons are showed at anterior side (black arrowheads). [file 12861_2021_242_MOESM2_ESM.jpg]

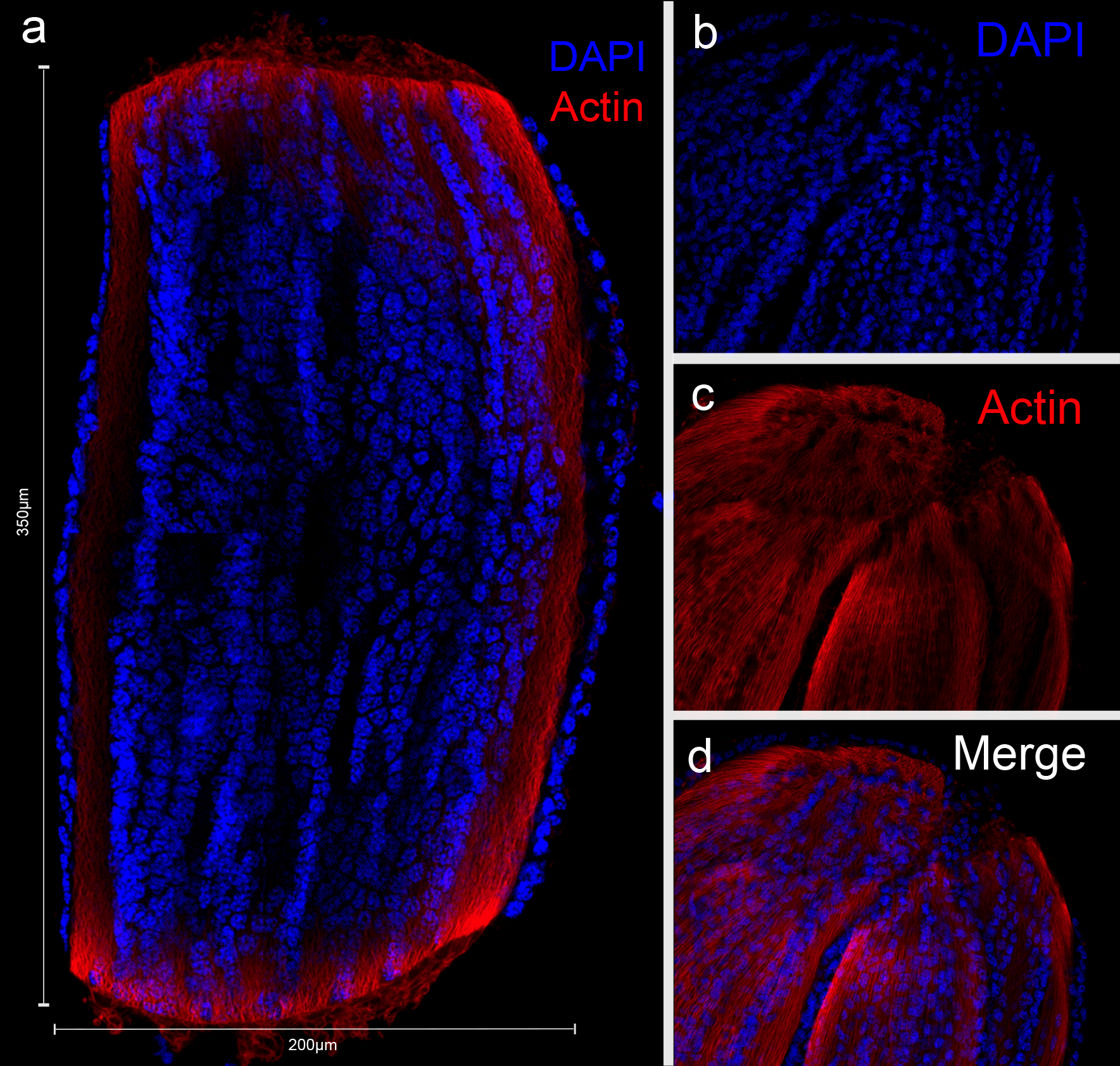

Supplement: Supplementary file 3 — Additional file 3: Fig. S3. Actin organization and distribution of nuclei in L4 IFM primordium. IFM primordia from L4 were stained with Rhodamine phalloidin and DAPI and representative optical slices are presented. a The nuclei form long rows between the actin filaments, that are along the primordium forming premyofibrils. b–d Upper view of L4 primordium showing five fascicles with premyofibrils. b Nuclei DAPI stained; c filamentous actin labeled with rhodamine phalloidin. d Merge. Scale bar, 100 μm. [file 12861_2021_242_MOESM3_ESM.tif]

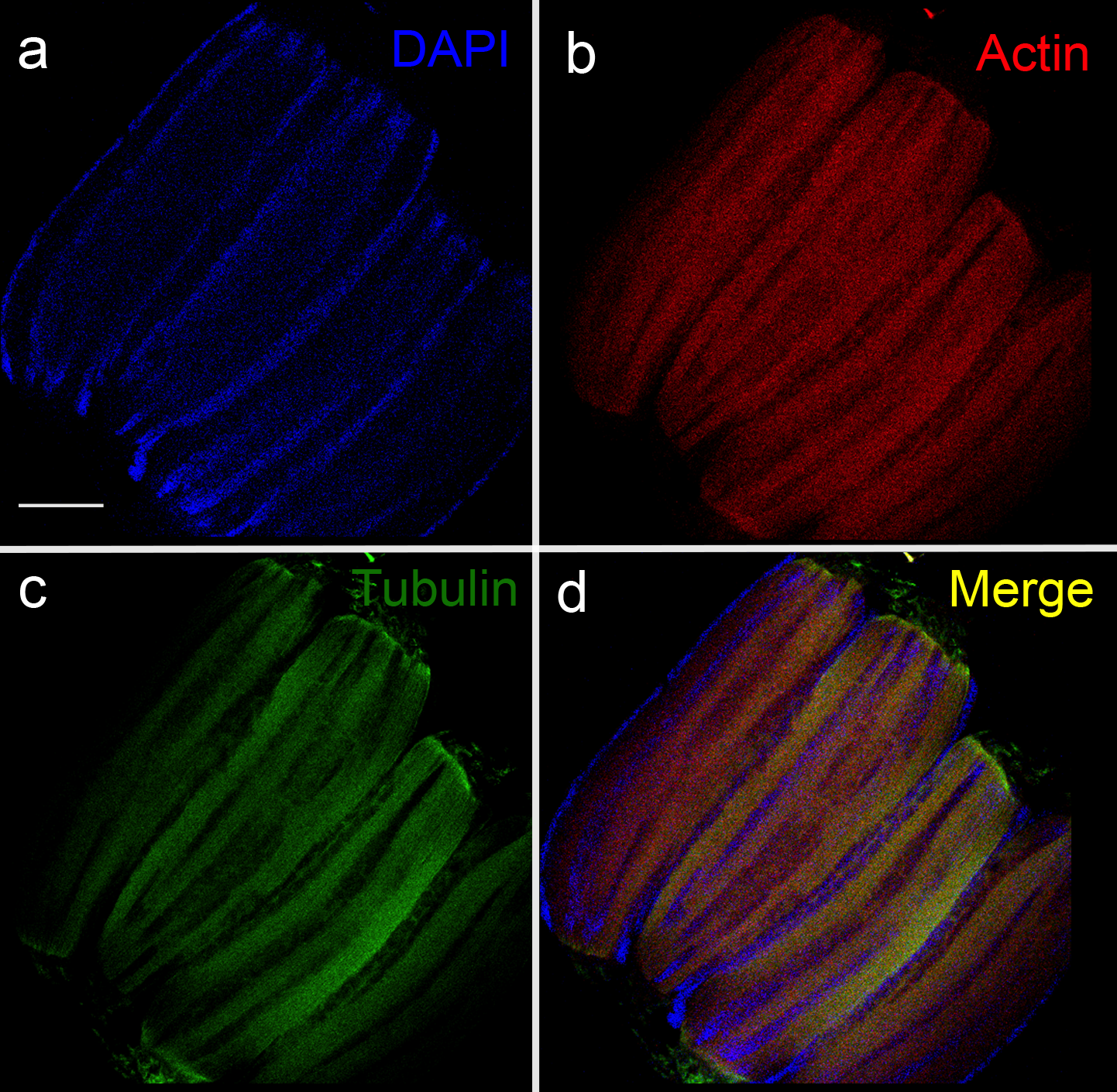

Supplement: Supplementary file 4 — Additional file 4: Fig. S4. Tubulin organization in L4 IFM primordia. Developing isolated primordia from L4 A. aegypti larvae were stained using a specific anti-tubulin antibody and rhodamine-phalloidin to label F-actin. a–d A representative optical slice is presented. a Nuclei forming rows. b Actin filaments along the myofibrils. c Muscle primordia showed a tubulin distribution pattern parallel to actin filaments. d Merge. Scale bar 50 μm. [file 12861_2021_242_MOESM4_ESM.tif]

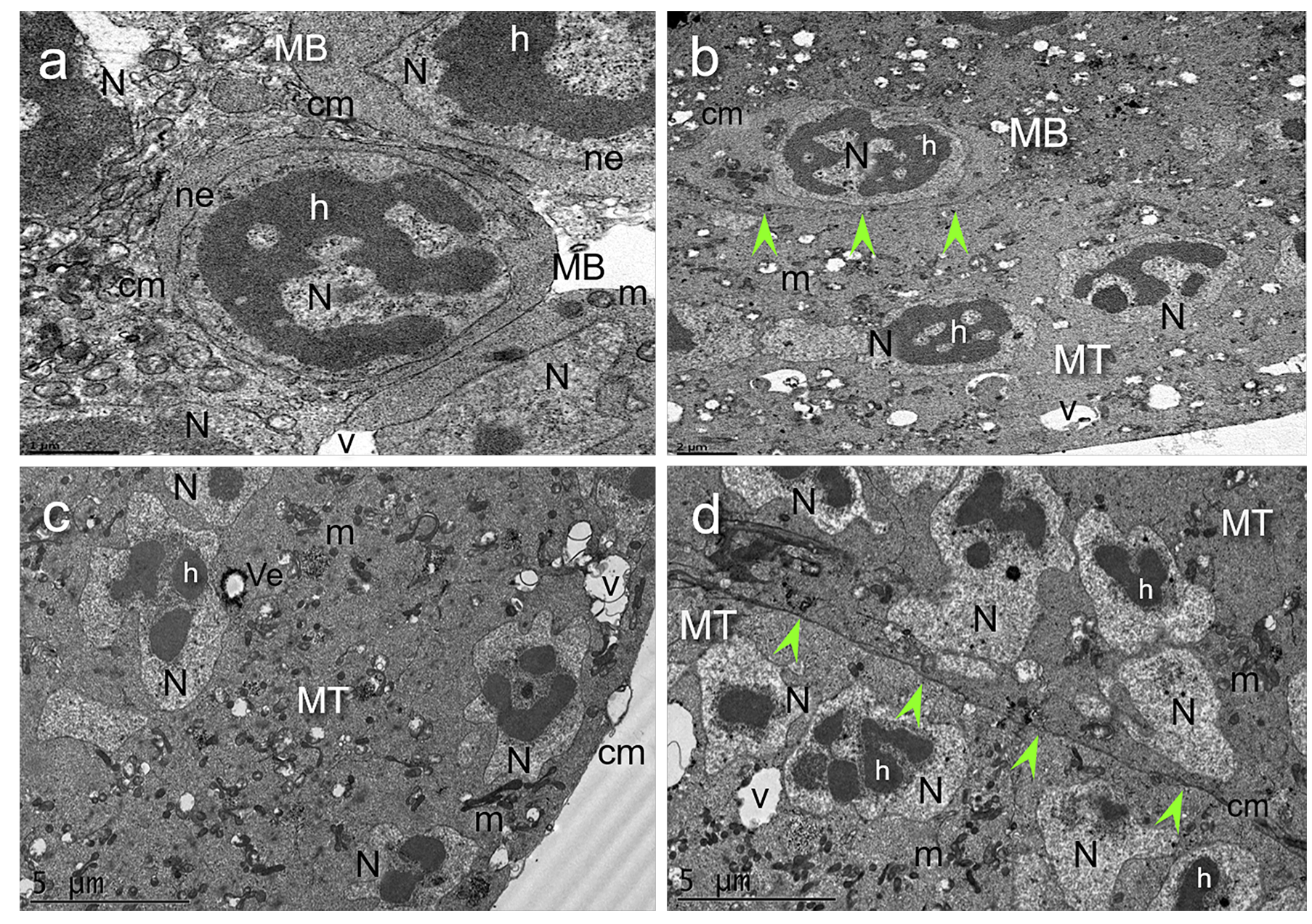

Supplement: Supplementary file 5 — Additional file 5: Fig. S5. Morphology of individual myoblasts and multinucleated myotubes. IFM primordia from late L4 instar were dissected and analyzed by TEM. a Individual myoblast. b–d Fused myoblasts forming multinucleated myotubes with nuclei aligned in rows and bordered by membranes (green arrowheads). In d, two multinucleated myotubes closely associated, lined by membranes were observed. All nuclei are pleomorphic with relaxed chromatin and abundant granular material. Many small mitochondria are present. MB, myoblast; MT, myotube; N, nucleus; h, heterochromatin; ne, nuclear envelope; cm, cell membrane; m, mitochondria; v, vesicles; ve, vesicle with electron-dense material. Scale bars: a 1 μm; b 2 μm; c, d 5 μm. [file 12861_2021_242_MOESM5_ESM.tif]
